# Editorial: Exosomes and exosomal miRNAs as biomarkers in infection with Mycobacterium tuberculosis

**DOI:** 10.3389/fcimb.2023.1239739

**Published:** 2023-07-26

**Authors:** Shamila D. Alipoor

**Affiliations:** Department of Molecular Medicine, Institute of Medical Biotechnology, National Institute of Genetic Engineering and Biotechnology, Tehran, Iran

**Keywords:** exosome, biomarker, exosomal miRNA, miRNA, tuberculosis

TB is still a major global health problem despite the superb diagnostic therapeutic technologies that are currently accessible in health medicine (World Health Organization (WHO), 2022).

Active TB was by far the most common infectious cause of death worldwide before the COVID-19 epidemic. However, the COVID-19 pandemic substantially hindered the detection of TB cases (McQuaid et al., 2021; Migliori et al., 2021; Lindmeier, 2022)

The End TB Strategy was created by the World Health Organization (WHO) to eradicate tuberculosis (TB) entirely by 2035.

By 2030, relative to 2015 levels, these objectives aim to reduce the number of TB cases and tuberculosis deaths by up to 90% (WHO).

Accelerating case finds and early TB diagnosis would be the major activities to achieve these aims, and the use of biomarkers is the primary approach in this regard.

In the study of tuberculosis, both pathogen- and host-based biomarkers have been extensively researched. These include hematologic markers, proteins, a number of metabolites, and signatures that incorporate many markers and have been found using unbiased “omics” discovery approaches (MacLean et al., 2019; Yong et al., 2019; Wykowski et al., 2021). However, the research findings are inconsistent, and there are still pitfalls and limitations in translating biomarker discoveries into clinical applications. Hence, more effort is needed to succeed in this field.

miRNAs are small non-coding RNAs that fine-tune complex biological processes by regulating the key proteins in molecular signaling networks. Due to their varying expression patterns in healthy, latent, and active TB populations as well as in different types of tuberculosis, these molecules have gained more attention as possible biomarkers in the TB field (Alipoor et al., 2016a; Alipoor et al., 2020).

In addition, miRNAs have determinant roles in the outcome of Mycobacterium tuberculosis (Mtb) infection (Alipoor et al., 2020). Mtb subverts the host miRNA network to modulate host cell signaling pathways favoring intracellular survival (Alipoor et al., 2017).

Mtb infection leads to host immune and metabolic repatterning, which enables Mtb to perturb the autophagy and apoptosis of infected cells and maintain their nutritional and energy requirements.

This process involves the modulation of host miRNAs that control the regulatory networks associated with cell metabolism and immunity in the infected cells (Alipoor et al., 2017; Alipoor et al., 2019).

The TB-dysregulated host miRNAs may be shuttled across the cells’ membranous organelles, such as exosomes.

Most cell types produce bioactive exosomes, which are 30-100 nm nanovesicles that carry a complex cargo of biomolecules from the original cell. Circulating exosomes are highly stable in biological fluids and therefore provide a great deal of information about the physiological and pathological status of the originating cell. These properties have fulfilled their promise as diagnostic biomarkers, enabling noninvasive clinical diagnosis (Alipoor et al., 2016b).

Numerous recent studies have shown the importance of exosomes and exosomal miRNAs in the fate of TB. It has been shown that the amount and composition of miRNAs packaged into exosomes (exosomal miRNAs) are different in infected versus uninfected macrophages, and also in the serum exosomes from TB patients versus healthy subjects (Hu et al., 2019; Lyu et al., 2019; Biadglegne et al., 2021). Exosomal miRNAs may be useful in the detection and monitoring of tuberculosis, according to these findings.

Despite the advances in understanding the content of Mtb and Mtb-infected host extracellular vesicles, our understanding of the biogenesis and role of extracellular vesicles during Mtb infection is still nascent. Furthermore, understanding the physiology and mechanisms of Mtb, and the mechanisms of orchestration of the host immune system against the bacteria can be interesting research fields to have a better understanding of the pathogenesis of the disease and to improve TB management.

In this Research Topic, we have gathered four studies covering these areas of research.

Mehaffy et al. have reviewed the intricate phenomena of tuberculosis and exosomes, as well as the variations in protein structure, physiology, and interactions with the human host. Extracellular vesicle (EV) capture, kinetics, and purification methods, as well as potential contributions to EV biogenesis in mycobacteria, are effectively discussed in this review.

Liang et al. have discovered a diagnostic model based on a 3-plasma miRNAs biomarker signature (hsa-miR-506-3p, hsa-miR-543, and hsa-miR-195-5p) that discriminates spinal tuberculosis (STB) from pulmonary tuberculosis (PTB) and other spinal diseases of different origins (SDD).

Fang et al. have shown the role of Rv0790c, a protein encoded by Mtb, in promoting the survival of the bacteria inside the cells. They observed that Rv0790c promotes intracellular mycobacterial life by suppressing cellular autophagy at an early stage following MTB infection. These findings aid in understanding the mechanism of Mtb evasion of host cellular degradation and hold the potential to develop new targets for the prevention and treatment of tuberculosis.

Tian et al. have reviewed the role of cytotoxic T lymphocytes (CTLs) in eliminating intracellular bacteria. They discussed the mechanism of CD4^+^/CD8^+^ CTL differentiation and formation, regulation of key transcription factors, and how CD4^+^ and CD8^+^ CTLs kill intracellular bacteria. The authors also describe the application and prospects of these cells in the treatment of intracellular bacterial infections.

Even with current progress in the fundamental research in the field of tuberculosis, we still need to deepen our understanding of the mechanisms of M. tuberculosis as well as the function of the host immune system to find suitable biomarkers and make a breakthrough in TB management, to ultimately meet the goals of the WHO to eradicate TB by 2035.

## Author contributions

The author confirms being the sole contributor of this work and has approved it for publication.
